# Supplementary figures and images for: In vivo clearance of nanoparticles by transcytosis across alveolar epithelial cells
Source: PLoS One. 2019 Sep 30;14(9):e0223339. doi: 10.1371/journal.pone.0223339 (PMC6768543; doi:10.1371/journal.pone.0223339)

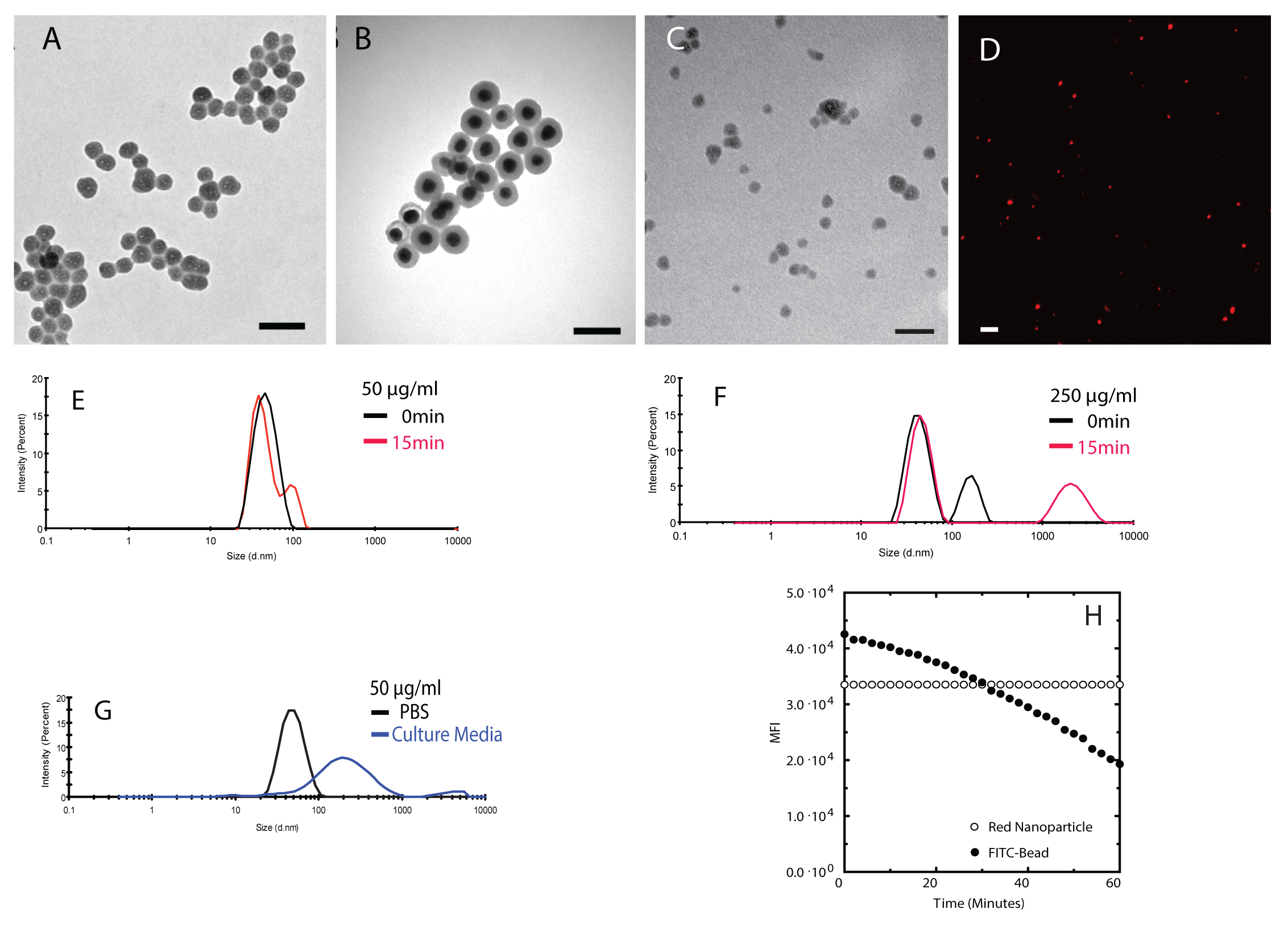

Supplement: S1 Fig — A: TEM of agglomerates of ø 50 nm red fluorescently labeled silica nanoparticle used for the intravital microscopy and B: ø 58 nm silica nanoparticle with 28 nm silver core used for electron microscopy of mouse lungs exposed at a low dose. C: ø 50 nm Cy7 infrared labeled silica nanoparticles used for isolated organ imaging. D: 2.5 μg of red silica nanoparticles were suspended in 2 ml culture media and the agglomerates were imaged using confocal microscopy. E-F: Hydrodynamic radii of agglomerates of ø 50 nm silica nanoparticle dispersed in phosphate-buffered saline (PBS) at 37°C, immediately following sonication (0 minutes) and after 15 minutes at 50 μg/mL (E) and 250 μg/mL (F), showing agglomeration is time and concentration dependent. G: Hydrodynamic radii of agglomerates of ø 50 nm-nanoparticle dispersed in phosphate-buffered saline (PBS) at 37°C (black) and cell culture media (blue). Note that the media drives agglomeration. H: In vitro, the red fluorescent nanoparticles used for the intravital microscopy do not bleach for a similar laser exposure as used for the intravital microscopy. For comparison, a FITC-labelled particle bleaches upon a similar exposure, as shown in the reduction of mean fluorescence intensity (MFI). (TIF) [file pone.0223339.s001.tif]

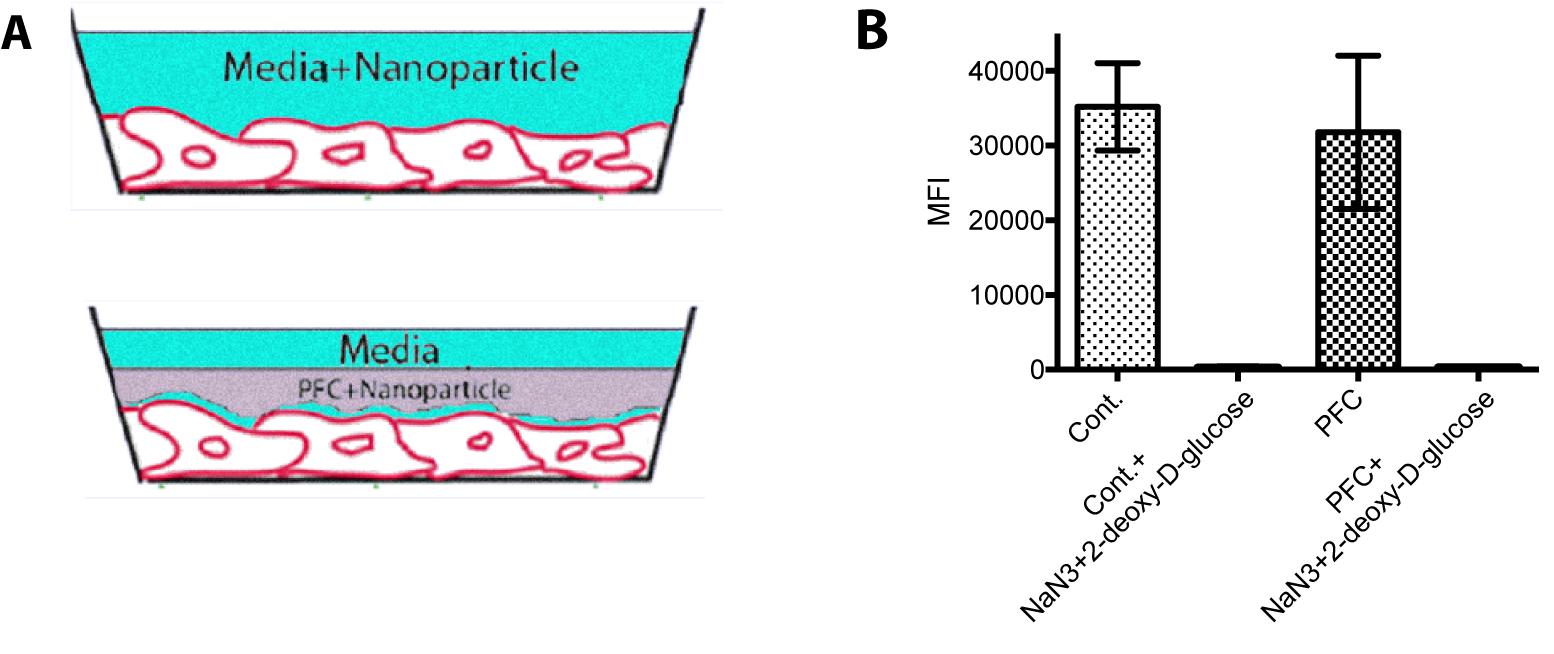

Supplement: S2 Fig — A: Sketch of the experimental setup to study the effect of delivering nanoparticles in perfluorocarbon (PFC) to lung epithelial (A549) cells on uptake as compared to delivery of particles in media. A549 cells cultured on 35 mm dishes and exposed to nanoparticles in media (Top) or suspended in PFC (bottom). B: Quantification of fluorescence (MFI) after 1h nanoparticle uptake in media (control), media + NaN3 + 2-deoxy-D-glucose (inhibition of endocytosis), PFC, PFC & NaN3 + 2-deoxy-D-glucose. (TIF) [file pone.0223339.s002.tif]

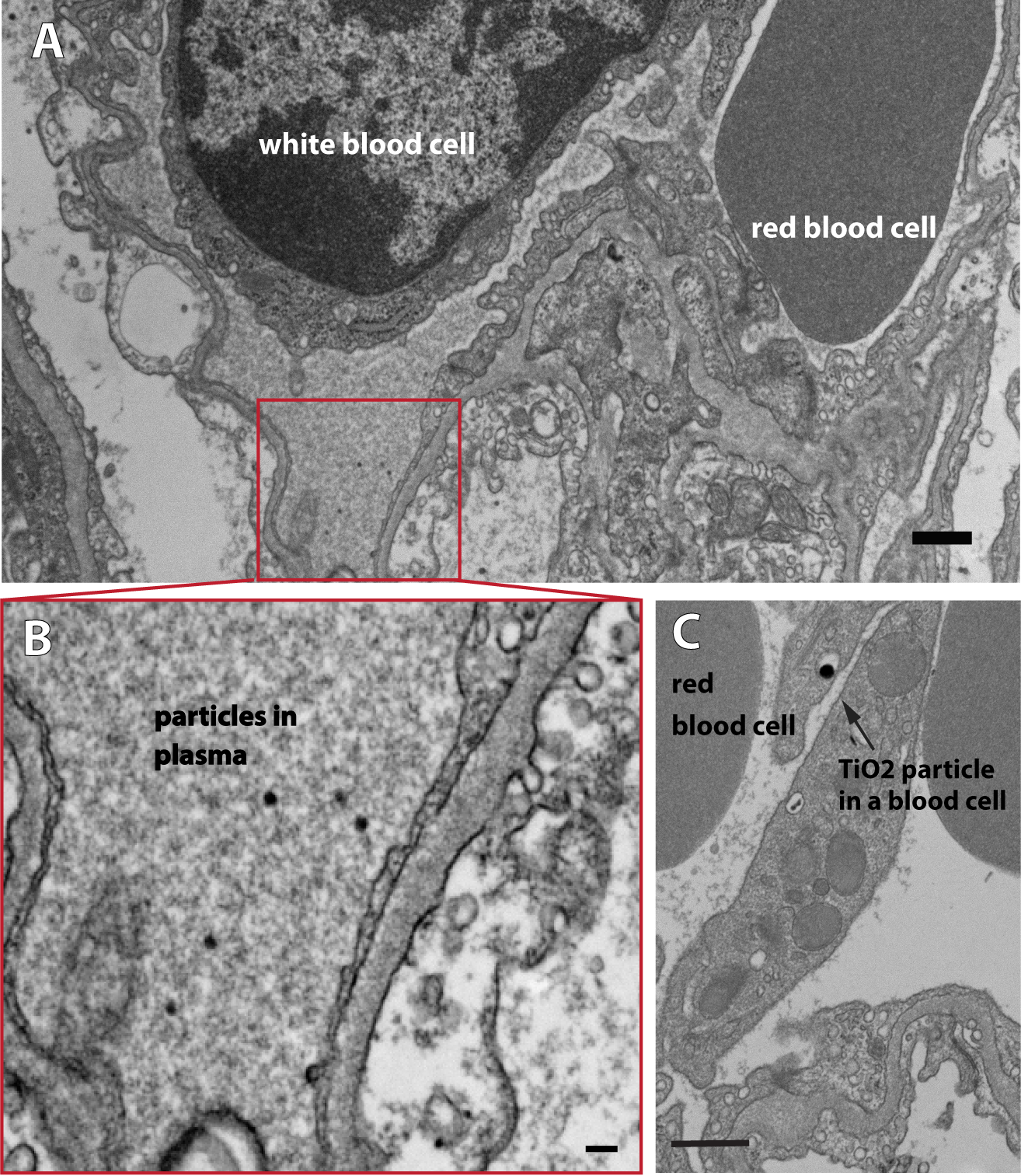

Supplement: S3 Fig — A-B: Mice were instilled with Cy7, infrared nanoparticles that have been used for mouse whole organ imaging. B: enlarged view of A showing evidence of particles in the plasma of the bloodstream. C: showing an example of a blood cell with a titanium oxide particle space bars: A & C = 500 nm, B = 100 nm (TIF) [file pone.0223339.s003.tif]

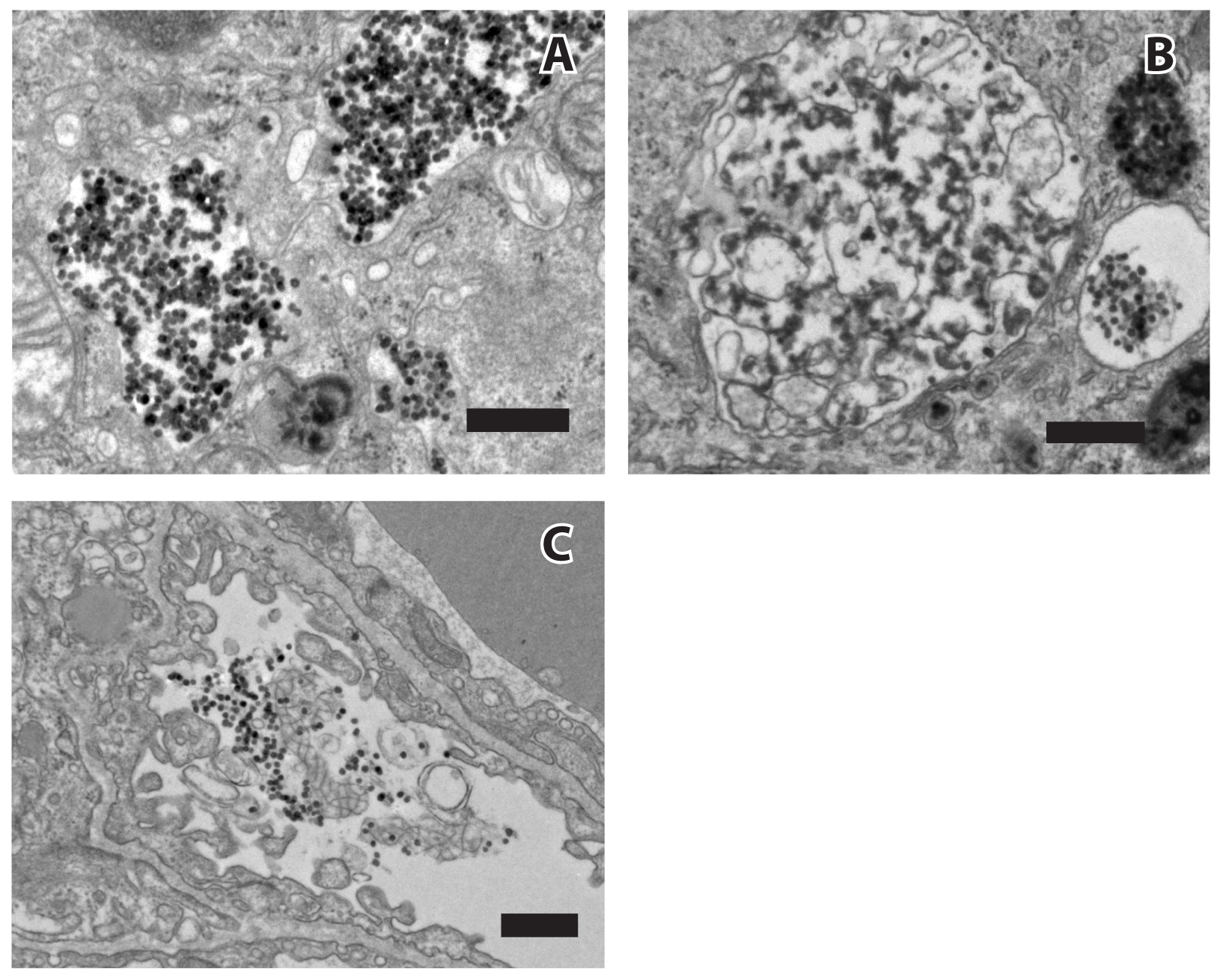

Supplement: S4 Fig — A: Magnification from Fig 3B, Electron micrographs of lung sections after instillation of 50 nm-silica nanoparticles into the lungs of mice. The particles inside an uptake compartment of type II alveolar epithelial cells are in dense agglomerates, similar to S1A Fig. The particles do not share the compartment with membranous structures. B: Magnification from Fig 3F. Particles inside uptake compartments of macrophages are dispersed and associated with the membranous structures of tubular myelin. C: Tubular myelin with entangled particles in the alveolar lumen looks like the tubular myelin with particles inside macrophages. space bars: 500 nm (TIF) [file pone.0223339.s004.tif]

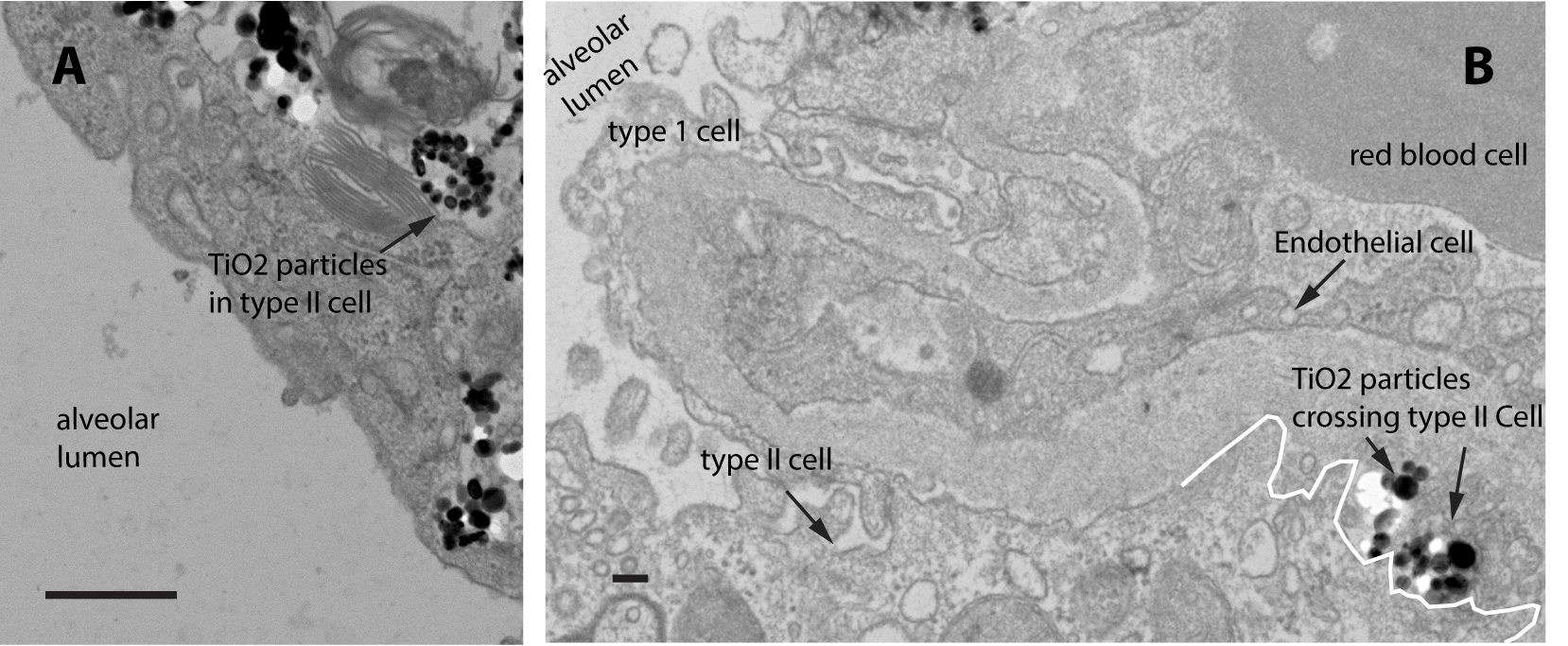

Supplement: S5 Fig — A-B: Mice (n = 2) were instilled with 3.8 μg/g body weight 1% Mn-doped TiO2 (ø<100 nm particles). A: shows TiO2 nanoparticles inside a Type II cell. B: shows an example of TiO2 nanoparticles having crossed the alveolar epithelium similar to the silica nanoparticles. space bars: A = 500 nm, B = 100 nm. (TIF) [file pone.0223339.s005.tif]
